# Conservation tools: the next generation of engineering–biology collaborations

**DOI:** 10.1098/rsif.2023.0232

**Published:** 2023-08-16

**Authors:** Andrew K. Schulz, Cassie Shriver, Suzanne Stathatos, Benjamin Seleb, Emily G. Weigel, Young-Hui Chang, M. Saad Bhamla, David L. Hu, Joseph R. Mendelson

**Affiliations:** ^1^ Haptic Ingelligence Department, Max Planck Institute for Intelligent Systems, Stuttgart 70569, Germany; ^2^ Schools of Mechanical Engineering, Georgia Institute of Technology, Atlanta, GA 30332, USA; ^3^ Biological Sciences, Georgia Institute of Technology, Atlanta, GA 30332, USA; ^4^ Chemical and Biomolecular Engineering, Georgia Institute of Technology, Atlanta, GA 30332, USA; ^5^ Division of Engineering and Applied Science, California Institute of Technology, Pasadena, CA 91125, USA; ^6^ Zoo Atlanta, Atlanta, GA 30315, USA

**Keywords:** conservation tech, human-centred design, AI4Good, Tech4Wildlife

## Abstract

The recent increase in public and academic interest in preserving biodiversity has led to the growth of the field of conservation technology. This field involves designing and constructing tools that use technology to aid in the conservation of wildlife. In this review, we present five case studies and infer a framework for designing conservation tools (CT) based on human–wildlife interaction. Successful CT range in complexity from cat collars to machine learning and game theory methodologies and do not require technological expertise to contribute to conservation tool creation. Our goal is to introduce researchers to the field of conservation technology and provide references for guiding the next generation of conservation technologists. Conservation technology not only has the potential to benefit biodiversity but also has broader impacts on fields such as sustainability and environmental protection. By using innovative technologies to address conservation challenges, we can find more effective and efficient solutions to protect and preserve our planet’s resources.

## 1. Background and motivation

The term ‘**conservation technology**’ was first proposed by Berger-Tal in 2018 [1] to broadly describe the use of technology to manage and conserve wildlife. While a commonly referenced example is unmanned aerial vehicles [2,3] (UAVs, also known as drones), there are many other conservation technologies, including camera traps [4,5], wildlife trackers [6,7], smartphone applications (apps) [8–10], devices for remote sensing and gathering geospatial data [11–13], and collection of environmental DNA [2,14–16]. Much of the existing technology uses modern hardware and software design processes to improve ongoing conservation efforts and initiate previously under-addressed efforts [1]. Some of the major goals of conservation technology are to improve outdated equipment, increase accessibility to tools and use modern technology to address conservation problems in entirely new ways. Conservation technology is being developed for animals in both natural environments and captive settings (e.g. foxes in urban settings and elephants in zoos, respectively) [17] and may also be applied to plants, habitats and geological phenomena.

Why have the last few years shown increasing interest in implementing both new and old tools in the conservation sector? The alarming rates of biodiversity loss amidst the current mass extinction [18] have driven demand for conservation technology. Since 1970, monitored populations of terrestrial, freshwater and marine vertebrates have plunged by an average of 69% [19]. With advancing technological revolutions over the past millennium, many scientists, engineers and other conservation stakeholders see conservation tools (CT) as critical methods for addressing ongoing conservation challenges.

Historically, the field of conservation technology has taken an opportunistic approach wherein stakeholders invest in developing technology for a specific, non-conservation need that is then applied to **wildlife** management [1], a prime example being the development of drones by the military. While **opportunistic technologies** certainly aid in wildlife management efforts, they tend to be expensive and less accessible to the conservation community. Consequently, there has been an increasing push towards purpose-driven technology designed in consultation with members of the conservation community [1]. Critically, technology is not considered to be proper conservation technology until its success for managing and conserving wildlife has been demonstrated.

Producing purpose-built technology requires a variety of skills that are typically beyond the scope of a single person, so establishing successful interdisciplinary collaborations is crucial. To properly synthesize these perspectives, conservation technology must establish the necessary bridges between the conservation community, technologists and policymakers [1,20]. However, these interdisciplinary collaborations are dependent on effective communication across domains, which can be difficult given differences in objectives and goals. While technology development and outcomes are often derived from an engineering design mindset, biological conservation is more hypothesis-driven and grounded in the scientific method.

Parachute science [21], where scientists ‘parachute’ into places for purposes of research or conservation but leave without a trace of acknowledgement or interaction with community members who made the work possible, is an unfortunately common phenomenon [22]. Researchers are frequently criticized for going to foreign, often less privileged, communities to gather data in their fieldwork and then leaving with their data alone without creating ties to the community. There are many forms of parachute science, sometimes called colonial science (leading to **colonization of conservation**) [23] or parasitic science [24]; for this paper, we define it as exclusion and ignorance of the knowledge of local communities. In the discussion of conservation science, local and indigenous knowledge is paramount to successfully implementing CT. Moreover, and arguably more importantly, without community inclusion and support, it is impossible to ensure lasting operational impact from research studies. Achieving real change from a research study requires local engagement.

Despite grants, training and other efforts towards these collaborations, the conservation community has encountered many solutions claiming to be universal-minded but lacking in necessary interdisciplinary knowledge and partners in respective fields. Inadequate communication between fields initially led the fauna and flora community to be distrustful of new technologies because many engineering solutions were poorly applied to serve the needs of conservationists, especially in natural outdoor situations. However, the pressing nature of the sixth mass extinction and climate change has made the necessity of interdisciplinary solutions evident and worth pursuing despite communication difficulties. With this expansion of interdisciplinary collaborations comes the realization that novel contributions to conservation technology can be designed in a variety of ways.

The term ‘conservation technology’ has received much criticism from the conservation community for the implication of requiring advanced technologies. While contributions can involve complex techniques such as machine learning to identify and track species [25], plenty of modern innovations involve simple devices such as chili pepper fences used to deter African elephants from damaging farmland [26].

It is for these reasons that we propose the term **conservation tools** instead of conservation technology. We believe the term ‘tool’ better encompasses the diversity of devices and methods used in this field. Rephrasing also intentionally includes indigenous solutions used in traditional conservation practices around the globe, which may not be accurately described as ‘technology.’

CT ideally should be built using **human-centred design** (HCD). Purpose-built technology in the hardware and software industry is considered collectively under the term HCD. HCD operates by using a design mindset that focuses on the **context of the use** of the idea. A common example is the difference between checkout interfaces in different environments. Purchasing a beverage at a bar versus at a supermarket serves a similar purpose but involves a different context for exchanging money. In each scenario, there are a set number of customers and a set number of items each individual may buy. For the bar, there are many customers, but few items, while the opposite is true of the supermarket. Thus the design of the technology and processes that enable the purchasing of goods are in very different contexts of use. We will apply the same logic to CT in using a **human–wildlife (fauna and flora)-centred design (HWCD)** approach.

A HWCD approach for CT requires consideration of not just the human interaction with the device but also the interaction between humans and fauna and flora. While ‘human–flora conflict’ [27] is used to describe interactions in urban, farming, or wild settings that can cause large amounts of harm to human interests [28], ‘human–wildlife interaction’ is broadly used to describe both positive and negative interactions. HWCD is not a new concept, as it has been implemented for millennia by indigenous peoples that live, interact and move with the land. For designers from non-indigenous backgrounds, it is essential to understand that true **indigenous design** is only possible if the primary designers of a technology solution are from the native indigenous lands where the solutions will be implemented [29]. To ignore indigenous or other community-derived knowledge is to create a solution with only partial expertise or knowledge of the problem; for this reason, we implore readers to understand that the effectiveness of your tool relies on the active collaboration of the community, scientists and engineers. As we go forward in this manuscript, it is paramount for authors to understand that the best tools are created by indigenous researchers, scientists and engineers working collaboratively as they are the most knowledgeable folks in the world about the conservation challenges non-indigenous members outside the community have imposed.

This manuscript is meant to be a starting guide to introduce the field of creating CT to those without experience. Next, we present a glossary of terms that also allow current practitioners of CT to understand the diversity of this field better.

## 2. Conservation tools vocabulary

As the conservation technology field has grown, it has adopted many terms from other fields to describe CT accurately. Unfortunately, many of these technical terms are domain-specific and can alienate stakeholders. For example, the term ‘wildlife’ can be restricted to undomesticated animal species within certain fields or scientific literature, but the definition prevalent in conservation and conservation technology is often generalized to include both animals and plants. We define many of the terms commonly used to describe CT and provide corresponding publications with more details on these specific terms (table 1).

**Table 1.** Terms used by different groups practising using advanced and new technology to develop CT and references to find more information about each term.

term	definition	reference
application programming interface (API)	a set of rules that allow applications and programs to communicate with each other	Boateng *et al.* [30]
back-end interface	the server and work behind-the-scenes to allow the user interface	Smith [31]
image classification	the computer vision process of predicting a class of one object to an image	Krizhevsky *et al.* [32]
colonization of conservation	the historical legacy that is conservation is performed by those that colonized the areas where the conservation is performed	Loss *et al.* [33]
conservation technology	an interdisciplinary field that works to design technology to help prevent the sixth mass extinction	Berger-Tal & Lahoz-Monfort [1]
conservation tools (CT)	devices that are made and developed to be applied to the conservation of wildlife	this study
context of use	a design thinking that takes the exact use of the device as the primary design component	Jacobson [34]
*ex situ* conservation	conservation of a species outside its original place (e.g. in a zoo)	Braverman [35]
fine-tuning	the computer vision process of taking a model that has been trained on one task and tuning it to make it perform a different, similar task	this study
front-end interface	the interface that the user sees, sometimes described as the user interface	Smith [31]
frugal science	the concept of creating scientific tools that are the most accessible possible in the form of cost and functionality	Byagathvalli *et al.* [36]
graphical user interface (GUI)	a digital interface where a user can interact with various components such as buttons or text boxes	Edler *et al.* [37]
human-centered design	a design thinking that takes the context-of-use of the exact devices as the primary design component	Jacobson [34]
human–wildlife centred design (HWCD)	using the human–wildlife interaction in the design process similar to that of human-centred design	this study
indigenous design	a design thinking that is designed by the indigenous population that is most familiar with the conservation and ecological initiatives	Nawrotski & Kadatska [38]
*in situ* conservation	conservation of a species at the original place (e.g. in the wild)	Braverman [35]
object detection	the computer vision process of detecting instances of semantic objects of a particular class or set of classes	Lin *et al.* [39]
object tracking	the computer vision task of taking a set of initial object detections, creating a unique identifier for each detection, and tracking each object over a series of time	Yilmaz [40]
object re-identification (Re-ID)	takes object detection one step further by matching a given object in a new environment to the same object in a different environment	Stewart *et al.* [41]
open-source solutions	solutions that are open access and solutions that are fully accessible by the public to re-create, re-design and re-invite	Lerner & Tirole [42]
opportunistic technology	devices that are built for a particular industry, such as camera traps designed for hunters, but used in a different purpose, such as biologists using camera traps for ecological surveys	Berger-tal & Lahoz-Monfort [1]
silver-bullet solutions	a one-size-fits-all solution that can address and solve any issue	Shaw [43]
self-supervised learning	a machine learning subset in which a model trains itself to learn part of the input from another part of data, often leveraging the underlying structure of the data	Hendrycks *et al.* [44]
supervised learning	a machine learning subset of problems where the available data has labelled examples	Russell & Norvig [45]
transfer learning	a machine learning method that uses a pre-trained model as a starting point for a model in a new task (i.e. it has already learned how to ‘see’ one set of things and will be trained again to get better focus on another set of things)	Zhuang *et al.* [46]
unsupervised learning	a machine learning subset of problems that analyses and clusters unlabelled data	Schmarje *et al.* [47]
wildlife	collective term referring to non-domesticated species of animals, plants and microbes, though sometimes restricted to just mean animals (particularly mammals and birds)	Usher [48]

Next, we present five case studies of CT that have been implemented worldwide. In each case, we emphasize the importance of identifying potentially affected communities when developing CT.

## 3. Discussion

**Figure 1.**
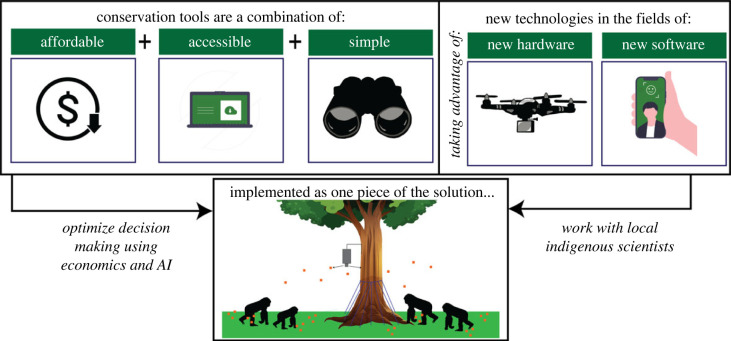
Visual abstract displaying the conservation tool framework discussed in this piece. Silhouettes created by Gabriela Palomo-Munoz and Undraw.co.

The core principles for CT are highlighted in figure 1 and are stated at the beginning of each case study. The following questions are discussed within each case study.

— What: what is its use?
— How: how is it used?
— Where: what are the use cases/how it helps?
— Why: future directions/open questions/etc.

We present case studies of CT representing both new and old technologies. Each of these solutions uses some measure of HWCD, with some employing frugal materials while others take advantage of hardware and software that have become more accessible in recent years. These case studies represent themes that the conservation community believes to be the most important tools for assisting in advancing conservation, according to the most recent state of conservation technology report [49]. Additionally, many of these solutions and case studies use several of the included principles for successful solutions. Computer Vision (Case study 3) often relies on advancing hardware technology (Principle 2) as well as advancing software (Principle 3), similar to that of eDNA and eDNA extraction methods.

### 3.1. Case study 1: open-source solutions

***Principle: solutions should be open-source* [*49*] *to promote accessibility and collaboration***

The increased use of **open-source** solutions, as opposed to proprietary ones, has emerged as a transformative trend in conservation technology [51–53]. We use the term ‘open-source solution’ to encompass hardware and software (or combinations of both) that are designed and developed under the principles of the open-source model. This philosophy seeks to make the design, blueprint or code of a solution freely accessible to the public. This is to allow anyone to use, modify, and contribute to the development of the solution [50]. Open-source solutions offer a myriad of benefits for conservation efforts. Firstly, they provide an accessible, affordable option for researchers and conservationists, breaking down financial barriers to technological utilization. Moreover, open-source tools enhance collaboration across disciplines, bridging the gap between engineers who develop technology and biologists who use the tools and interpret the data. The AudioMoth acoustic logger serves as a prime example of the open-source paradigm, demonstrating its practical application and successful adoption in many conservation initiatives.

*What is its use?* In the case of what is the use of open-source solutions, we can reframe this as: what are open-source solutions suitable for? Effective wildlife management decisions often require abundant data on the animals and plants. Compared with other methods, acoustic monitoring devices can substantially increase monitoring coverage of animals both in terms of land area and recording time [54–56]. These remote monitoring devices are minimally invasive and can be deployed into areas of interest to listen for the calls of co-located animals and abnormal activity without causing disruptions to the environment [54–56]. Such aspects make this technique a powerful environmental monitoring strategy applicable across many environments and contexts. Until recently, however, acoustic monitoring systems have been too expensive and complex for mass implementation in the scientific community.

The AudioMoth acoustic logger revolutionized this narrative when it came onto the market in 2017, costing only $49.99 USD [57], one-tenth the price of comparable commercially available audio recording devices (in 2017). Beyond the low cost, the device is small (the size of a credit card), energy-efficient, and records both human-audible sounds and ultrasonic frequencies.

**Figure 2.**
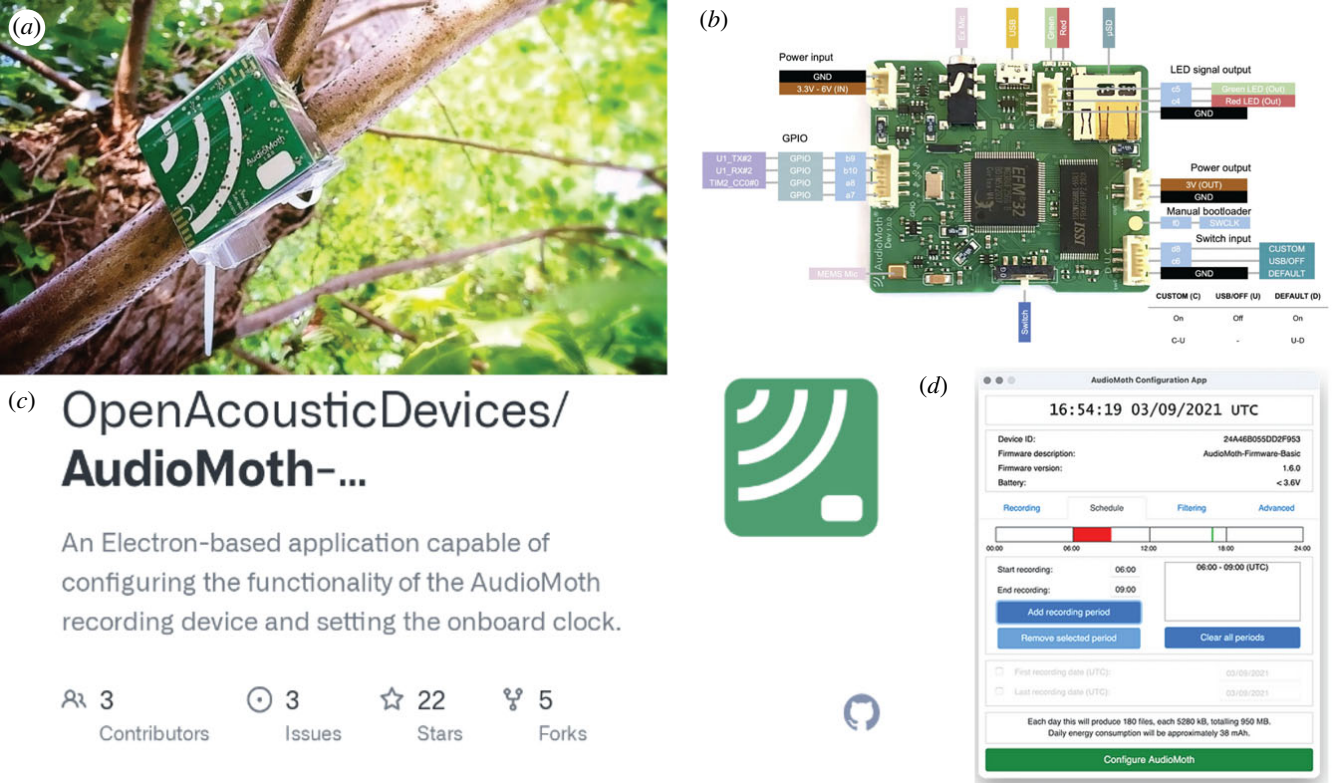
(*a*) AudioMoth, (*b*) open source printed circuit board that Open Acoustic Devices (https://www.openacousticdevices.info/) included on the website, (*c*) open source code for controlling and interpreting data from the AudioMoth via GitHub, (*d*) online and app-based user-interface for AudioMoth users. Images were taken from the Audio Moth website with permission.

*How is it used?* The AudioMoth was created by two PhD students with the intention of increasing scientific accessibility (figure 2*a*) [57]. This device was developed with a HCD approach, integrating modern technology in both software and hardware, with a keen understanding of its end-users—biologists, who often have different needs and skill sets compared with computer scientists or engineering researchers. The developers have made design files, such as the circuit board, housing schematics and software, freely available. This transparency allows users to modify their devices or even construct their own, tailoring the tool to their specific needs.

It should be acknowledged that the AudioMoth is still a sophisticated piece of technology, and building or modifying it may be beyond the technical capabilities/infrastructure of many conservation practitioners. The developers address a key challenge in conservation technology, ensuring devices are still accessible while ensuring sustainable production. They accomplish this via group purchase campaigns to get them fabricated and distributed. Besides handling the fabrication process, bulk order production has the added benefit of reducing per-unit costs. Groupgets is a service that, similar to crowd-funding, is a crowd-purchasing of electronics. AudioMoth and other devices operate where they open a Groupgets (https://groupgets.com/) campaign, and a total number of people have put in an order (say 500) the order is closed and these items are distributed. This entire process is described further in Hill *et al*. [57].

Once the device is in hand, implementation can be expedited via a **graphical user interface (GUI)**. This GUI can be easily downloaded onto most personal computers and allows users to quickly set up the device as a scheduled recorder without any specialized knowledge of the embedded software running on it. Because the software and firmware running on the device are also open-source, the developers encourage users to edit the source code for more advanced customization of their devices, such as case-specific filtering to actively classify sounds of interest [53].

*What is the use case?* Thanks to its enhanced accessibility and options for customization, the AudioMoth has found diverse applications in the field of conservation. It has been used to monitor animal populations [58], track migrations [59], identify poaching activity [53], detect sounds underwater [60] and even discover new species [53].

*What is the potential?* AudioMoth’s success exemplifies the power of open-source solutions in conservation, broadening the range of applications and the adoption of tools. Its open-source approach, combined with a focus on biologist end-users and a sustainable production model, has provided a viable pathway for creating versatile, affordable and customizable tools.

Since the release of the AudioMoth, many other products have come to market with comparable features at similar price points. The Song Meter Micro [61], for example, assists with the increase of scientific accessibility of bio-acoustic data collection; it costs $249.00 per unit and operates in a wide range of environments, including glaciers in Northern Greenland [62]. It is essential to realize that the Song Meter Micro is not open-source, but the price of this and other similar devices to the AudioMoth have probably been driven down thanks to the open-source products on the market. The AudioMoth allows the user only to have to use the provided **front-end interface** if needed but also can use customization of the **back-end** for these advanced cases. Other devices and technologies sometimes employ application program interfaces (API), which allow communication between different computer programs.

A new journal publication type is leveraging the future of open-source hardware through publications. These journals currently include the *Journal of Open Hardware, The Journal of Open Engineering* and *HardwareX*. These journals require all submissions to include complete information for all hardware and software included in the device [63]. It should, however, be mentioned that some of these publishers, including Elsevier, are for-profit publishers.

### 3.2. Case study 2: environmental DNA

***Principle: solutions should take advantage of increasing hardware technologies***

DNA analysis is a well-established scientific tool for an ever-expanding scope of biological studies [64]. An enormous challenge in the use of DNA for purposes of conservation is that traditional methods of DNA collection require biological samples such as urine, hair, skin or other tissue [65]. Traditional biological methods have historically required the restraint, capture, or rapid collection of fresh DNA samples that can be either logistically infeasible or actively at odds with observing organisms in the wild. If organisms are rare or secretive, it may not be possible or logistically feasible to get samples from them. Thomsen and Willerslev (2015) reviewed using eDNA as an emerging tool in conservation [66]. One of the primary challenges they highlighted in conservation is the trade-off between the invasiveness of studies and data collection. Applications of eDNA are reducing the need for invasive studies and enabling locating and monitoring of creatures too rare or secretive, or sensitive to disturbance. It may not be possible, logistically or ethically appropriate for traditional survey methods.

*What is its use?* Environmental DNA (eDNA) allows for the analysis of diets, geographical ranges, population sizes, demographics and genetics, as well as the assessment of the presence or absence of species at sites. These can be quantified with eDNA collected from samples such as faeces left behind, hairs snagged on vegetation, or water samples in the case of aquatic environments, and later analysed for different genetic information. Using eDNA benefits the conservation space as being a holistically non-invasive DNA extraction method, making it very repeatable. The techniques for collecting eDNA still require biological sample collection methods, but the samples can be in much lower concentrations and then extracted through post-sample collection methodologies.

*How is it used?* Environmental sampling of biological sampling is a previously developed tool that has been applied in several applications. This includes recently using waste water for coronavirus monitoring [67]. This technique of biological sampling from the environment in the form of eDNA has been adopted by wildlife conservationists, which allows using DNA samples found in the environment for understanding endangered species in their natural environment using only DNA samples. The novelty of this tool is its ability not only to detect DNA information about animals but its applicability across various environments, including land, sea and even in polar ice [66]. As a tool, eDNA is helpful in various conservation and ecological fields, but this solution has had a significant history of colonial-style parachute science [68]. It is important to note that while solutions and technology like this can be leveraged, they must be thought of in the HWCD framework. Working with local and indigenous communities is crucial because eDNA is not the sole solution, and additionally on-ground conservation work is necessary for the long-term conservation of wildlife [69].

*What is a use case?* One of the first documented uses of eDNA was in 1992 when Amos used shed skin from cetacean mammals species to inform a population analysis [70]. Although not considered as conservation technology at the time, this was one of the first applications of non-invasive eDNA for biological conservation and population assessment. Now eDNA is used to monitor not just populations but to catalogue local biodiversity of fishes [71], manage reptile populations [72] and forest conservation [73] using the interface between remote sensing and eDNA.

*What is the potential?* Despite its broad range of potential applications, eDNA is not a universal solution for all situations, mainly because the methodology is complex in terms of sampling and acquisition of data. Sample processing of eDNA often requires sample preparation in very precise chemical and biological processes with humans executing precise tasks. As such, eDNA is prone to the same human errors as other laboratory-based risks, including contamination, biased results and interpretations, or even as simple as not having adequate reference databases for identifying DNA sequences for all regions or applications.

These pitfalls do not discount eDNA as an example of using new technological advances and scientific progress to advance conservation practices. Tools such as this are continually being improved and innovated, and eDNA devices are becoming cheaper and more accessible for scientists. This field has been expanding in the past years with increasing establishments of DNA *barcodes* that permit the identification of species using online DNA databases [74]. DNA barcoding takes shortened segments of DNA and allows for rapid species identification. This rapid DNA identification can be used for rapid identification of fish by specific species having a DNA fingerprint that can be rapidly checked to help prevent mislabelling [75].

### 3.3. Case study 3: computer vision

***Principle: solutions should take advantage of increasing software technologies***

Machine learning is the science and art of developing computer algorithms to learn automatically from data and experience [76]. Computer vision is a subfield of machine learning in which computers and systems are trained to extract meaningful information (aka ‘see’) from images, videos and other inputs. Computer vision lets computers understand visual inputs [77] and offers many benefits over traditional image review and annotation.

*What is its use?* While humans have been *trained* during their lifetime to identify objects, understand their depth, and see their interactions, computer models require thousands of labelled images to teach machines to recognize new scenarios with the efficacy of a human observer. With hardware advances and algorithms designed for lower-resource devices, computer vision has become less expensive and more accessible. From only being able to work on stationary super-computers, computer vision is now standard, with all computation and data storage being performed on small devices in the field like laptops and cell phones [78–80]. Users of computer vision applications today include, but are not limited to, (i) iPhone users to unlock their phones with their faces, (ii) drivers of self-driving cars, and (iii) traffic enforcers who use red-light traffic cameras.

**Figure 3.**
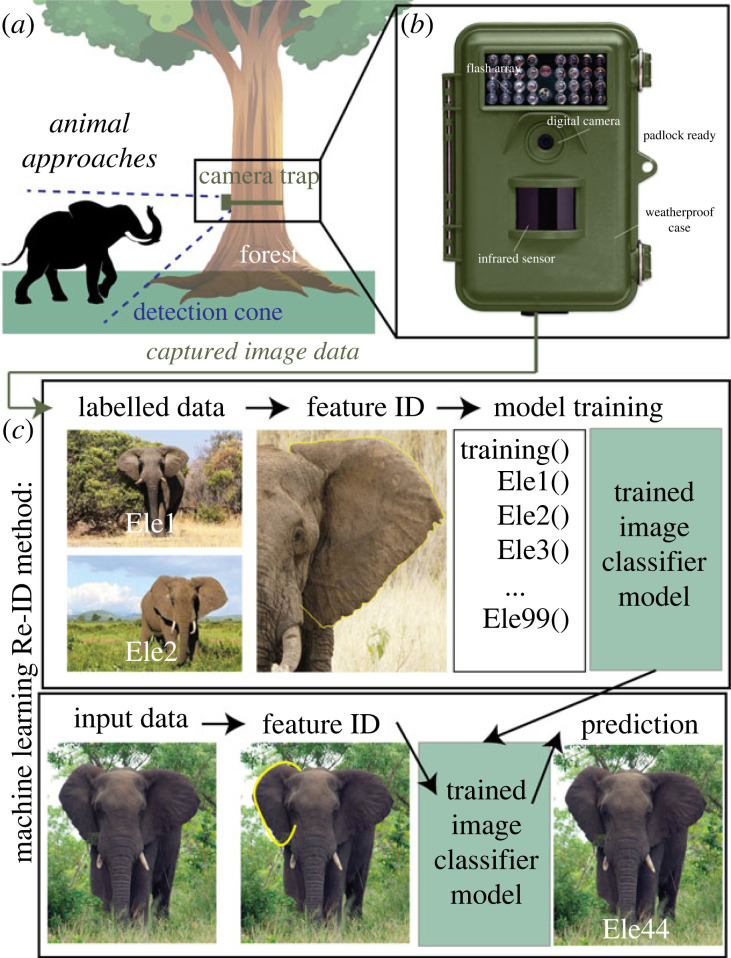
Basic camera trap set-up. (*a*) A camouflaged camera trap is often placed on a tree or a pole. (*b*) Camera trap model. It is equipped with a motion-triggering sensor, a digital camera and a memory card. When an animal passes in the region of interest, the camera captures photos/video at a specified frame rate of the animal. (*c*) The figure was made using a dataset from LilaBC [81] and images from Flickr.

*How is it used?* Conservationists use camera traps to capture images of wildlife. A typical camera trap apparatus is shown in figure 3*a*. A camera is placed in a region of interest. This could, for example, be a known location where animals go, such as near a water source. It passively collects information about what goes through that region. Camera traps collect data over a specified period, either writing to an external hard drive or pushing data to a cloud-hosted framework. Camera traps often include infrared and/or motions sensors that can identify warm-bodied or moving objects. When an animal triggers the sensor, the camera records (writes images to memory), as shown in figure 3*b*. Previously, humans have had to sift through these images to extract meaningful information about the animals. More and more, computer vision techniques are being applied to this imagery to help scientists detect, track, classify and re-identify (recognize) individual animals, among other things [82,83]. A machine learning model can either train on the camera trap’s imagery to learn and recognize patterns particular to that the data, or a pre-trained machine learning model can be used out of the box to predict these features of interest (figure 3*c*).

Traditionally, computer vision has used classical **supervised** machine learning algorithms (algorithms that need human-labelled data). These algorithms let the model understand identifying characteristics of the animals within the regions of interest (for example, colour histograms, texture differences, locomotor gait etc.; figure 3*c*) [25]. The model can learn to detect and classify wildlife species from those characteristics in the images. Two key use cases of this include classifying animal species (classification) and recognizing and identifying individual animals (**re-identification or Re-ID**). In these tasks, extraction of the foreground of the image is an important pre-processing step to focus the model on the animal of interest. For example, the first step in classifying urban wildlife is often to crop the image to focus on the animal and not to focus on cars, trees and other environmental features [84]. The model could then take these cropped images and classify them as different species types (i.e. squirrels, dogs, coyotes etc.). Alternatively, as seen in several urban wildlife monitoring projects, computer vision has been used to crop humans out of images and ignore empty images [85].

**Figure 4.**
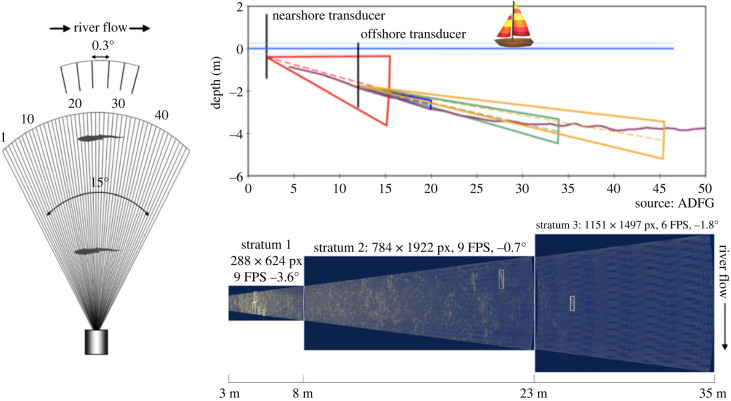
Sonar camera arrangement: here are some diagrams of the camera deployment. On the left, you can see the camera shooting out multiple acoustic sonar beams—they are used to pick up fish in high resolution. The closer the fish are to the camera, the higher their resolution is. In the top right, you can see how these sonar cameras are placed to ‘see’ all areas where the salmon might swim. There are two sonar cameras—the one with the red triangle only captures one field of view; the one with the three narrow triangles oscillates between capturing three different strata (20 min at one, 20 min at the second, 20 min at the third). The bottom right image shows what these three strata images look like when combined. The white boxes are the annotated fish swimming through the stream. Images have been provided from the Alaska Department of Fish and Game and Caltech [86].

*What is a use case?* Recent advances in hardware have allowed computer vision to expand to underwater locations. The Caltech Fish Counting task leverages sonar cameras placed in rivers to detect, track and count salmon as they swim upstream [86]. The set-up of these cameras within rivers is illustrated in figure 4. They cannot rely on infrared sensors, so they capture images continuously across a specified period. Fisheries managers review the videos and manually count the number of salmon. Caltech researchers are working on automating this with computer vision [86].

*What is the potential?* Computer vision has led to a set of technologies that can aid wildlife conservation across terrestrial, aquatic and laboratory environments. Using computer vision as a tool can help solve limitations in manual data analysis by saving time and by limiting external bias. Processing large amounts of data quickly allows ecologists to then identify ecological patterns, trends etc. in their scientific space and facilitates quicker lead times on field observations. Their science, then, informs ecological actions and goals. Integrating computer vision into wildlife conservation is dynamically automating animal ecology and conservation research using data-driven models [25].

### 3.4. Case study 4: game theory and optimization

***Principle: economics and artificial intelligence should be leveraged in conservation challenges to optimize decision-making***

Artificial intelligence is actively being used to combat wildlife threats. When designing CT like sensors, one key challenge is where to place them in an animal’s ecosystem to collect relevant data. Researchers are looking into ways to leverage artificial intelligence methods to optimize conservation/resource planning and policy-making. One such field in computer science that differs from computer vision is the use of game theory for more effective data collection. Game theory is a collection of analytical tools that can be used to make optimal choices in interactional and decision-making problems. The use of game theory for conservation has only recently become a field of study.

*What is its use?* In non-mathematical terms, optimization is the study of how to make the best or most efficient decision given a particular set of constraints. In probability theory and machine learning, the multi-armed bandit problem is one type of optimization problem in which a limited set of resources must be split among/between competing choices to maximize expected gain. This problem is a subclass of a broader set of problems called stochastic scheduling problems. In these problems, each machine provides a reward randomly from a probability distribution that is not known as *a priori*. The user’s objective is to maximize the sum of the rewards. These techniques are commonly used for logistics (routing) coordination and financial portfolio design, though they have also been adapted to be used for modelling nefarious actors and optimally countering them. In wildlife scenarios, biologists often have to use few tools to collect data in a vast environment, hundreds of square kilometres. The use of optimization strategies has recently begun to help ecologists and biologists pinpoint locations to collect data descriptive of a sizeable ecological habitat effectively.

*How is it used?* *Patrol Planning.* Wildlife poaching and trading threaten key species across ecosystems. Illegal wildlife trade facilitates the introduction of invasive species, land degradation and biodiversity loss [87]. Historically, park rangers have recorded where poachers have struck. However, most national parks have a limited supply of park rangers. They often are limited to driving, walking, or biking around the parks. Several parks have repositories of historical data detailing poaching locations identified in the past. This data can be used to predict likely poaching threats and locations in the future. Work has been done in the game theory and optimization space to leverage machine learning (on the historical data) and optimize multi-modal (i.e. driving and walking) patrol planning. Ultimately, parks and wildlife conservation organizations want to find the optimal answer to the questions, ‘How should I organize my patrols?’ and ‘How will adversaries respond?’ [88]. This optimization technique provides them with a way to answer those questions directly.

*Economic modelling.* Additional researchers, including Keskin and Nuwer, are working toward understanding the *economics* behind these wildlife threats. Poaching is an additional income source for individuals in rural communities who may rely primarily on tourism for income. If these communities cannot rely on tourism, they may focus on wildlife trafficking, as those species are prevalent near them [89]. A review of wildlife tracking [90] focusing on operations and supply chain management recognized four challenges that limit preventative measures:

1. the difficulties of understanding the true scale of illegal wildlife trade from available data;
2. the breadth of the issue—trafficked animals are used for food, status symbols, traditional medicine, exotic pets and more (this requires the policy remedy to be multifaceted), and sometimes illegal wildlife trade operates in countries with corrupt governments or limited infrastructures for law enforcement and monitoring;
3. illegal wildlife traders are geared towards undetectable operations, especially from financial institutions; and
4. illegal wildlife trade is considered less serious than other trafficking, i.e. human, drugs and weapons.

There are several suggested ways to apply research in supply chain operations toward combating illegal wildlife trade [90]. These include: bolstering data through CT such as satellite data, acoustic monitoring, eDNA, news scraping and finding online markets; strengthening data detection and prediction through network analysis and understanding data bias; modelling the problem as a network interdiction problem to see how to disrupt the supply chain network; more effective resource management and reducing corruption. By analysing the complex supply chain and operations behind illegal wildlife trade, Keskin *et al*. [90] illuminated a more clear picture of each location/scenario individually, which allows an informed and targeted response to prevent illegal wildlife trafficking [32].

**Figure 5.**
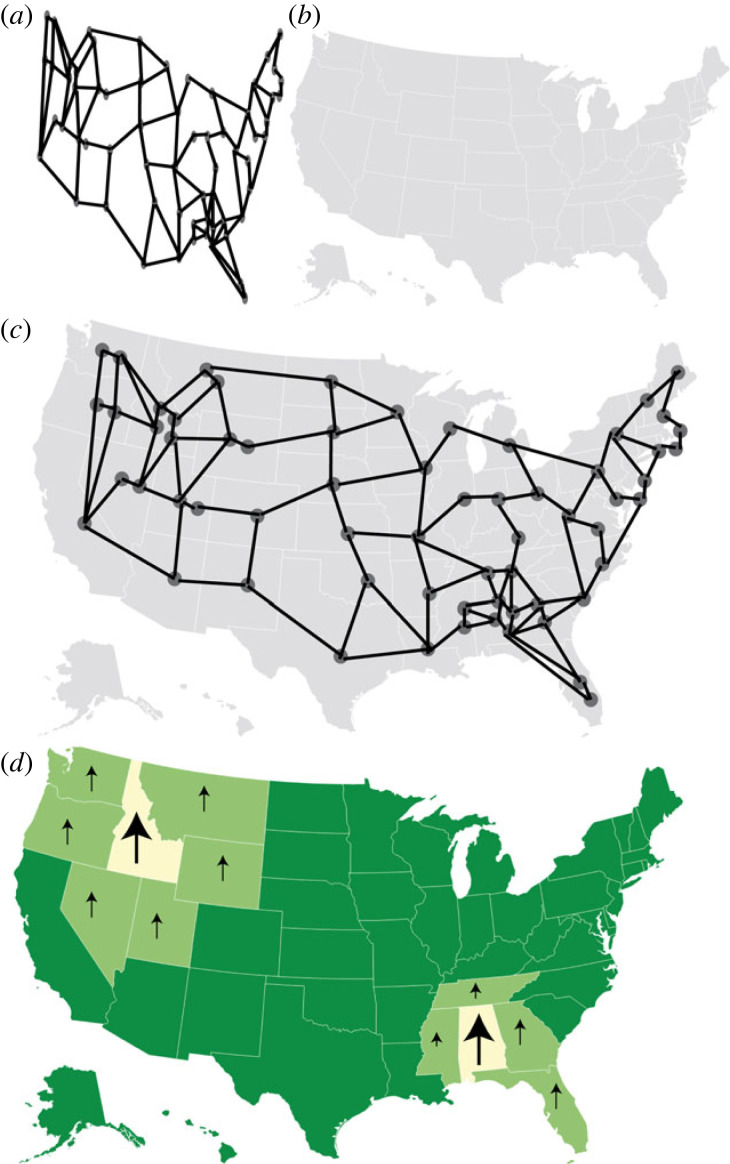
Using game theory and optimization for conservation practices. (*a*) Data mapping of a conservation issue to determine which states' conservation funding is most important. (*b*) Raw map of the USA. (*c*) Overlapped image of the clustering depicted in (*a*) with the raw map of the USA. (*d*) Data-interpreted map displaying large arrows in the states where the most conservation is needed with smaller arrows (in light green) displaying states where clustering is beginning. Images made using DataWrapper.

*What is a use case?* Evidence from parks in Uganda suggests that poachers are deterred by ranger patrols, illuminating the increased need for robust, sequential planning [88]. Computer science economists have worked on adversarial modelling to demonstrate poachers’ deterrence to patrols and other poacher behaviour patterns [88]. An illustration of poaching patterns with increased patrols is shown in figure 5.

Researchers working at the Jilin Huangnihe National Nature Reserve in China first used machine learning to predict poaching threats and then used an algorithm to optimize a patrol route. When rangers were dispatched in December 2019, they successfully found 42 snares, significantly more than they had found in previous months and patrols [91]. Combining machine learning and optimization techniques, therefore, has proven to increase the efficiency of patrol planning and can be expanded to more conservation management applications as well.

*What is the potential?* Applying optimization techniques across conservation-oriented tasks will provide insight and better resource usage to historically under-resourced applications and programs. In addition, these optimization techniques and economically focused viewpoints can prompt organizations and governments to identify and quell issues more efficiently. Programs can best use the limited resources they have and do so in an efficient data-driven manner. This can, in theory, be scaled to any resource-limited situation, too. Those with camera traps, for example, can study where to best place them to capture the most data-rich images. Those with limited AudioMoths, similarly, can study where to place them to ensure optimal and most realistic acoustic captures.

### 3.5. Case study 5: frugal solutions

***Principle: solutions can be simple and should not be over-engineered***

In contrast to many of the previously discussed case studies, a conservation tool can be as simple as a brightly coloured cat collar. This case study highlights the need for viable and accessible solutions, providing an example that fails to fit the technocentric vision of conservation technology that has taken hold. Advanced technology, while offering increased functionality, often comes with barriers to adoption such as high implementation cost or specialized knowledge requirements for utilization. These barriers are especially condemning for conservation practitioners operating in resource-limited environments. Even in developed regions, the cost and complexity of solutions can deter widespread adoption and utilization.

A potential strategy for increasing the adoption of CT is to design them as frugally as possible. This practice has been termed **frugal science** [36] and is subtly different from the Do-It-Yourself and Free and Open-Source Hardware movements in that it is solely focused on repurposing everyday items to create low-cost and straightforward devices. While frugal science has primarily been used to reduce the cost of medical and bioengineering equipment [92–94], its driving principles are uniquely suited to conservation efforts.

*What is its use?* Domestic cats kill 1.3–4.0 billion birds per year in the US alone [95]. This makes them one of the nation’s most significant anthropogenic threats to wildlife, yet very little counter-action has been taken. Potential methods of controlling this issue, such as enforcing indoor-only policies for pet cats or eradicating or neutering all feral cats, are often unrealistic or met with public resistance [96]. A brightly coloured collar presents a frugal solution to this problem. It has been found that cats wearing a Birdsbesafe collar ($11.99 USD) killed 19 times fewer birds than without [97], thus offering a passive and cost-effective approach to conservation.

*How is it used?* The collar consists of a frill of brightly coloured fabric that attaches to a standard breakaway collar and is easy to apply and safe for the cat. It’s bright colours alert birds and small mammals to the cat’s presence, reducing their predatory effectiveness. Notably, no technology is required. Far less costly and controversial than alternative measures, this low-cost solution allows domestic cats to remain outdoors while significantly reducing their threat to wildlife.

*What is a use case?* Consider a community where many residents own cats and let them roam outdoors. Despite awareness campaigns about the impact of cats on local wildlife, changing human behaviour proves challenging. Here, the brightly coloured cat collar offers a practical and cost-effective solution. Residents can easily adopt this frugal solution without significantly altering their or their pets’ routines. The collar, being simple to use and affordable, can be widely distributed, even within a large community.

*What is the potential?* Financing the development and implementation of conservation technologies is a significant challenge. While support from philanthropic organizations or technology organizations such as Google Earth, Bezos Earth Fund or AI4Good from Microsoft can provide initial funding, these opportunities are often limited and may not ensure long-term adoption.

Frugal solutions like the cat collar demonstrate a promising alternative. Their simplicity and low cost can facilitate broader implementation, making them particularly effective in resource-limited communities. Moreover, by focusing on repurposing everyday items to create low-cost and simple devices, the frugal science methodology can help broaden conservation efforts more generally. This approach not only reduces the financial barriers to adoption but also makes CT more accessible to a wider range of users. Frugal solutions can be crucial in advancing conservation efforts by prioritizing simplicity and cost-effectiveness.

## 4. Conclusion

***Conservation tools vary but are united in their potential to aid conservation***

**Figure 6.**
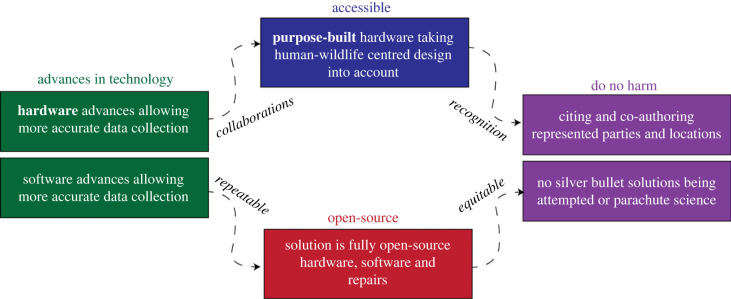
Process that goes into designing and using technology to develop new CT. Bolded terms are principle design components of conservation tool creation.

There is no single solution to the many challenges in conservation. CT are designed to be a part of a community’s toolkit to help conserve and protect wildlife, and we discuss the key themes that make them successful in figure 6. As these case studies show, CT are not meant to solve all problems, but they can be useful in contexts where previous methods are too onerous or costly. Developers of CT must understand that their designs need to be user-friendly for conservation practitioners and be viewed as a resource rather than a complete solution for addressing biodiversity decline. The most effective solutions are those that are realistically implementable and consider the context of human–wildlife interactions in the design process. In this paper, we review five case studies of specific CT that are advancing wildlife conservation. When examining these tools, it is important to consider the context in which they are used and the specific conservation issues they are addressing.

To develop effective CT, biologists, computer scientists and engineers must collaborate and apply their expertise. These interdisciplinary teams must also work with community members who have a deep understanding of conservation challenges. The wide range of perspectives and challenges addressed through these partnerships allow CT to take many forms. We highlight five key characteristics of successful CT. Open-source and accessible solutions like AudioMoth offer opportunities for crowd-sourcing and additional improvements, as well as the ability to adapt existing frameworks to similar problems. Technologies from other fields can be repurposed in innovative ways to benefit conservation, such as using eDNA to reduce the invasiveness of data collection techniques. Existing software like computer vision can also be applied to the conservation field to streamline and expand data analyses. Successful CT are not limited to biology, engineering and computer science; they can also benefit from non-traditional fields like maths for identifying ideal collection sites. Finally, not all solutions need to be high tech to be effective. Simple solutions, like cat collars with bells to protect birds, can also be effective CT.

In this paper, we aim to provide a foundation for future conservation tool creators by reviewing case studies of successful tools and highlighting key themes. These case studies demonstrate the diverse range of approaches that can be taken in conservation technology, from simple cat collars to complex machine learning and game theory methodologies. By drawing on the expertise of interdisciplinary teams that include biologists, computer scientists, engineers and community members, we can develop practical tools that address the unique challenges of each conservation context. As we work to conserve and protect wildlife, it is essential to remember that CT are just one part of a larger toolkit and should be integrated into traditional and indigenous approaches to conservation. Through this review, we hope to inspire the development of innovative solutions to address the pressing needs of biodiversity conservation. Ultimately, conservation technology is essential for addressing the challenges of biodiversity preservation and promoting sustainable solutions for human–wildlife interactions.

## Data Availability

This article has no additional data.
